# Nature relatedness as a potential factor to promote physical activity and reduce sedentary behavior in Ecuadorian children

**DOI:** 10.1371/journal.pone.0251972

**Published:** 2021-05-20

**Authors:** María José Molina-Cando, Samuel Escandón, Delfien Van Dyck, Greet Cardon, Deborah Salvo, Florian Fiebelkorn, Susana Andrade, Cristina Ochoa-Avilés, Ana García, Jorge Brito, Mario Alvarez-Alvarez, Angélica Ochoa-Avilés

**Affiliations:** 1 Bioscience Department, Faculty of Chemistry, Universidad de Cuenca, Cuenca, Azuay, Ecuador; 2 Department of Movement and Sports Sciences, Ghent University, Ghent, Belgium; 3 Prevention Research Center, Brown School, Washington University in St. Louis, St. Louis, Missouri, United States of America; 4 Didactics of Biology, University of Osnabrück, Barbarastrasse, Osnabrück, Germany; 5 Postgraduate Program in Immunology, Federal University of Bahia, Salvador, Bahia, Brazil; 6 Faculty of Philosophy, Carrera de Pedagogía de la Actividad Física y Deporte, Universidad de Cuenca, Cuenca, Azuay, Ecuador; 7 Physical Activity and Sports Pedagogy, Universidad Politécnica Salesiana, Cuenca, Azuay, Ecuador; Linneaus University, SWEDEN

## Abstract

**Background:**

Nature relatedness can be associated with health-related outcomes. This study aims to evaluate the associations of nature relatedness with physical activity and sedentary behavior.

**Methods:**

A cross-sectional study involving 9–12 year old children living in Cuenca, Ecuador, was conducted between October 2018 and March 2019. Questionnaires were used to evaluate physical activity (PAQ-C), out-of-school sedentary behavior, nature relatedness and wellbeing. Associations of nature relatedness with physical activity, and sedentary behavior were evaluated using linear regression models or tobit regressions adjusted by age, sex, school type, wellbeing, and body mass index z-score.

**Results:**

A total of 1028 children were surveyed (average age:10.4±1.22 years, 52% female.). Nature relatedness was positively associated with physical activity (β = 0.07; CI 95%: 0.05–0.09; p < 0.001) and non-screen-related sedentary leisure (β = 3.77 minutes; CI 95%: 0.76–6.68; p < 0.05); it was negatively associated with screen time (β = -5.59 minutes; CI 95%: -10.53–0.65; p < 0.05).

**Conclusions:**

Findings suggest that nature relatedness is associated with physical activity and some sedentary behaviors among Ecuadorian school-age children. The promotion of nature relatedness has the potential to improve health.

## Background

Physical inactivity and sedentary behavior (SB) are important risk factors for cardiometabolic diseases and type 2 diabetes [1]. Sitting time and TV viewing have been identified as independent risk factors for premature mortality [2]. Specifically in youth, regular physical activity (PA) and spending less time invested in SB have several benefits on health, such as improvements of physical fitness, bone health, weight status, social relationships, and mental health [3]. Moreover, daily PA in children and adolescents improves life satisfaction; this correlation is cumulative and bidirectional [4].

PA and SB during childhood are complex behaviors influenced by individual and environmental factors [5]. Age, sex, attitudes, habits, motivation, subjective norm, knowledge and beliefs, among others, are critical individual determinants of PA [6, 7]. On the other hand, family, school, community, social traditions, and the built environment are important environmental factors of influence [8]. Access to green spaces and participation in outdoor activities have several benefits, including higher levels of PA, and social and nature interaction [9]. However, few studies have examined the role of the natural environment on PA among children in low- and middle-income settings (LMIC). Nature relatedness has recently been proposed as a potential influencing factor for PA in children [9].

Nature relatedness is defined as the cognitive, affective, or experiential connection between humans and the natural environment [10]. The biophilia theory claims that human beings are irremediably linked to nature [11]. Research carried out among adults has consistently documented associations between nature relatedness with wellbeing, mood, cognition, pro-environmental attitudes, and ecological behaviors [12]. Recently, a study conducted among UK adults found that nature relatedness was associated with higher levels of use of green spaces and with a higher likelihood of meeting PA guidelines [13]. Positive feelings about nature might influence attitudes toward outdoor recreation, which in turn enhances the intention to visit and perform PA in such areas [14]. The available evidence demonstrates that children are experiencing a “nature-deficit disorder” [15]. Recent trends show that screen-based SB is replacing outdoor activities among children, which in turn may result in reduced PA and nature relatedness [16].

To the best of the authors’ knowledge, the relationship between nature relatedness with PA and SB has not been investigated in children. Besides, research on PA and SB and their determinants in LMICs, particularly in the Andean Latin America region, are scarce. Available data for these settings often have methodological limitations such as the selection of non-representative samples, use of unvalidated tools, lack of data on children, and inclusion of individual-level correlates only (i.e., sex, age, socioeconomic status) [17]. Identifying the correlates and/or determinants of PA and SB is a prerequisite for the design and implementation of setting- and culture-appropriate interventions and programs to promote healthy behaviors. Consequently, this study aims to identify the association of nature relatedness with PA and SB among children in Latin America. More specifically, we focus on a representative sample of children in a modern Andean metropolis of Ecuador.

## Materials and methods

### Participants and setting

A cross-sectional study was conducted in the urban and peri-urban area of Cuenca, located in the Andean Highlands of Ecuador. Cuenca is the third largest city of Ecuador with a population of 505,585 inhabitants and an area of 214 square kilometers [18]. It is an emerging and sustainable city based on its high quality of life indicators such as the provision of basic services, health, air quality, and security to its residents [19]. Cuenca has 332 public and private elementary schools located in the urban and peri-urban areas [20]. Public schools comprise tuition-free governmental schools and subsidized schools (monthly fee: 0 to 29.9 USD). Private schools are not subsidized (monthly tuition range: 30 to 425 USD) [21]. Classes in private elementary schools are held in the morning (07H00–12H45). Due to the high number of pupils, most public schools offer a double-shift-school system: morning (07H00–12H45) and afternoon (12H45-18H45) shifts [21].

#### Sampling and procedures

This study is part of the REDU-EDPA project, which aims to model the interactive effects of individual and environmental factors on school children’s health.

**Fig 1:**
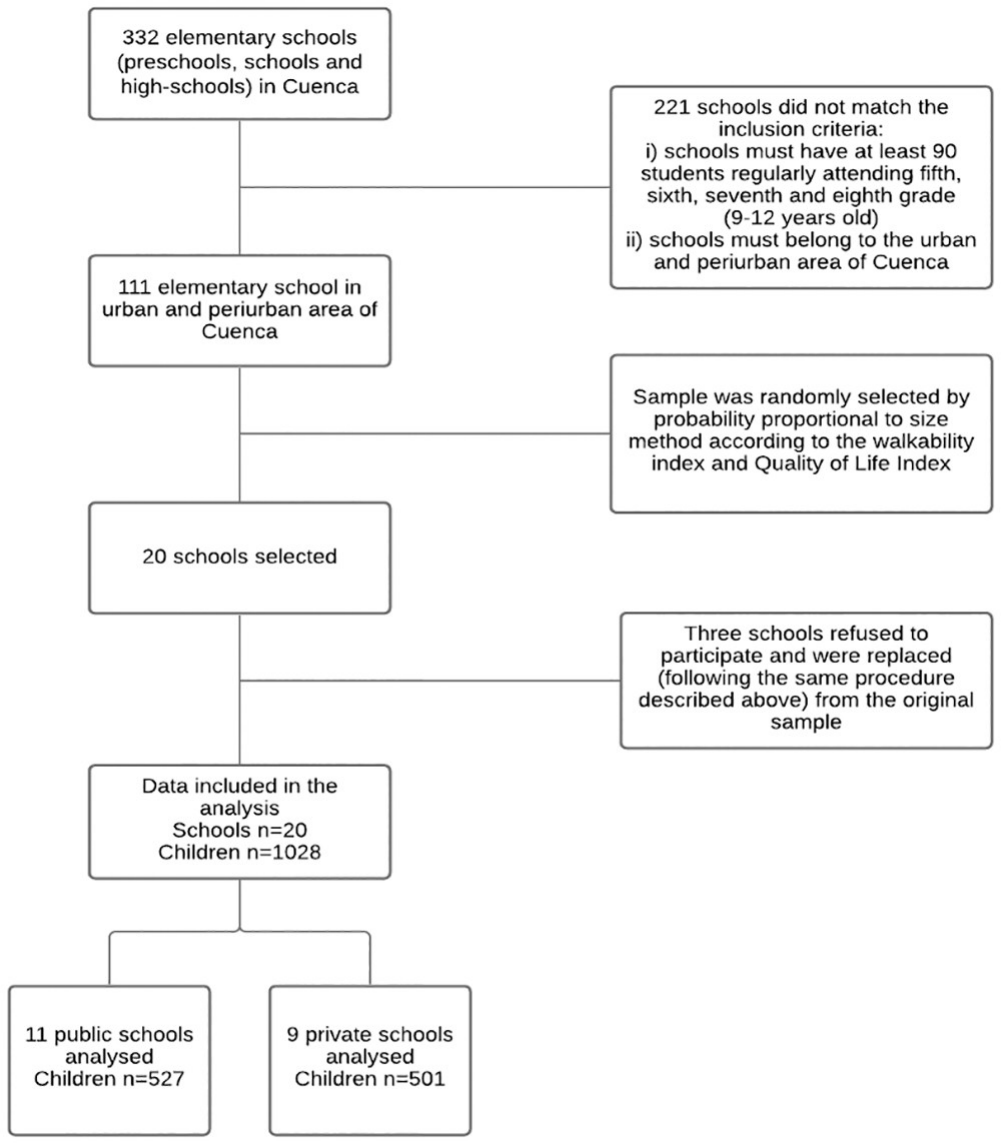
Flowchart for a sample selection of schools and participants.

Data were collected between October 2018 and March 2019. A cluster random sampling was determined, with schools as the primary sampling units (Fig 1). Eligible schools for inclusion i) were located in the urban/peri-urban area and ii) had at least 90 children aged 9–12 regularly attending fifth to eighth grade.

Twenty schools from a total of 111 that fulfilled the inclusion criteria were randomly sampled following a probability proportional to size procedure. Schools were selected according to their neighborhood walkability index and socioeconomic status (SES). The school neighborhood walkability index was calculated following the methodology proposed by the International Physical Activity and the Environment Network [22]. School neighborhood SES was estimated by means of the Quality of Life Index (QoL) (High vs Low QoL) according to the level of satisfaction of basic needs (housing characteristics, basic services, educational level, and access to health services) in the neighborhood, where zero represents complete lack of basic needs, one complete coverage, and >1 a quality of life above meeting basic needs [23]. Three schools initially selected refused to participate. These schools were replaced following the same procedure described above.

From each selected school, 90 randomly selected students were invited to participate, considering previous acceptance rates in similar studies (*n* = 47 students * 20 schools = 940 students) [24]. Parents/guardians of selected participants were invited to meetings to explain the project procedures and obtain informed consent. Consent forms were sent with the children to the parents/guardians who did not attend.

Children with disabilities or conditions that might influence their regular PA (i.e., Down Syndrome, congenital heart defects, musculoskeletal disorders, chronic diseases, cerebral palsy, hypothyroidism) or without a signed written consent were excluded. The study’s final response rate was approximately 47%.

The study was statistically powered at 80% for detecting associations between individual and environmental factors with the outcomes of interest and involved a cluster-random sample of 1028 school children aged nine to twelve years.

### Ethics statement

The study protocol was approved by the Ethics Committee of Universidad San Francisco de Quito (No. 2017-090E). Written consent was obtained for each participant and their parents/guardians, respectively. All the staff members involved in data collection signed a confidentiality agreement. Students with identified risk factors (e.g., underweight, overweight, or obesity) were referred to specialists from the Universidad de Cuenca (i.e., nutritionists, physicians). At the end of the project, the results of the weight status, physical activity levels and sedentary behavior were sent to the parents/guardians by email. In addition, basic recommendations based on the available WHO guidelines were provided together with a telephone contact number for any queries about the results.

## Measurements

Data was collected at school by trained field workers with tablets during school hours following standardized procedures, using the KoboToolbox application [25].

### Outcome measures: Physical activity and sedentary behavior

PA was assessed using the Spanish version of the Physical Activity Questionnaire for Older Children (PAQ-C) [26]. The PAQ-C is a self-administered seven-day recall instrument that provides a summary PA score. Reliability of the PAQ-C has been reported in Colombian children with an intraclass correlation coefficient (ICC) > 0.73 and a Cronbach’s alpha of 0.83 [27]. The PAQ-C collected data based on nine items. Item one estimates children’s leisure-time activity, items two to eight focus on PA in physical education classes, recess, lunch, immediately after school, during the evenings and weekends; finally, item nine takes the average of the intensity of PA performed every day during the last week. Each item is scored between one (low PA) and five (very high PA), and the average of the nine items are used to derive a total activity score where the highest levels represents higher PA levels (range: 1–5).

SB outside school-hours on a typical weekday was measured using a Spanish version of the self-report measure of SB used in the “international Healthy Environments and Active Living in Teenagers study” (iHealt) adapted to local conditions [28]. High reliability of the questionnaire was observed in a sample of 200 children aged 8–12 years-old who were not included in the current study (ICC: 0.66 for total time spent in SB). The total time spent in SB outside school hours during a habitual school day was calculated by summing the minutes reported in specific types of SB: screen time behavior (watching TV, DVDs, using a computer for doing homework), non-screen-related sedentary leisure (time spent in reading for fun a book or magazine). SB is reported as total SB (min/day), screen time (min/day), and non-screen-related sedentary leisure SB (min/day).

### Exposure: Nature relatedness

**Fig 2:**
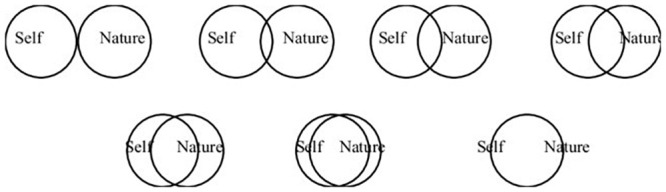
Inclusion of nature in Self scale based on Schultz (2002).

Nature relatedness was assessed through the Inclusion of Nature in Self scale (INS) [29]. The scale consists of seven pairs of overlapping circles, labelled “self and nature” (Fig 2). The graphical-item is based on an adaptation of the “Inclusion of Another in Oneself Scale” with test-retest reliability of 0.84 over four weeks [30]. The INS has been used with children in Ecuador in a previous study and proven to have a similar or greater consistent correlation compared to other measures of self-nature connection [31, 32]. The least overlapping circle receives a score of one, and the most overlapping a score of seven; therefore, the higher the value, the higher the nature relatedness.

### Covariates

Sociodemographic data (age, sex, type of school), anthropometric measures (BMI z-score), and wellbeing were included as covariates. The type of school (i.e., public or private) was used as a proxy for the participants’ SES. In Ecuador, children from families with lower socioeconomic indexes are concentrated in public schools, while children with higher socioeconomic indexes tend to attend private schools [33].

Weight was measured using digital scales (SECA 803 Germany) and recorded with an accuracy of 100g. Height was recorded to the nearest mm using portable measuring devices (SECA 213 Germany). Children’s privacy was guaranteed by choosing female staff for girls and male staff for boys. Participants wore light sportswear for measurements (underwear and shorts). Two measurements were taken, and a third measurement was needed when the deviation between the two readings for weight and height was greater than 2% [34]. Body mass index (BMI [kg/m^2^]) was calculated from weight and height. BMI-for-age-z-scores were estimated using the who2007.ado macro for Stata [35].

Wellbeing was estimated using a validated Spanish adaptation of the “Satisfaction with Life scale for children” (SWLS-C) [32], which is one of the most frequently used scales for measuring life satisfaction [36]. Our adapted version of the SWLS-C (Cronbach’s α = .70) included the following five items on a 5-point Likert scale (“totally disagree = 1” to “totally agree = 5”): (1) In most ways, my life is how I want it to be, (2) The conditions of my life are good, (3) I am satisfied with my life, (4) So far I have gotten from my life the things I consider important, and (5) If I could live my life over, I would change almost nothing. A mean score (1–5) was calculated, where the highest levels represent greater levels of wellbeing.

### Data analysis

Descriptive statistics are presented as percentages and means ± standard deviations. Age, PA, SB, wellbeing, and nature relatedness were compared by sex and type of school subgroups using independent sample t-tests. Post-estimation graphic methods were used to evaluate the regression models. In case the observations did not meet the criteria, a tobit regression was applied. The association of nature relatedness with PA, total SB, and screen time SB was evaluated independently using linear regression models with PA, total sedentary time and screen time SB as dependent variables (outcomes) and nature relatedness as the independent variable (exposure). To estimate association between nature relatedness and non-screen-related sedentary leisure, a tobit regression model was performed. All models were adjusted for sociodemographic data, BMI-for-age-z-score, and wellbeing. The models were additionally adjusted for PA when associations for total sedentary time, screen time SB, and non-screen-related sedentary leisure were the outcomes, and for total SB when PA was the outcome. All the analyses were adjusted for the cluster design (using the “svy” command in Stata) with schools as the primary sampling units. All analyses were performed at a 5% significance level using Stata V.12.0 [37].

## Results

### Descriptive statistics

A total of 1028 children from 20 schools participated in the study. Fifty-five-percent of the schools were public, and 60% had less than 1000 children attending regularly (Table 1). Half of the schools (n = 10) only had morning shifts and their most commonly reported fees were less than $30 (55%). The mean age of the participants was 10.4±1.2 years, 52% were female, and 51% attended public schools (Table 2). The mean score for PA was 2.7±0.5 and was higher in boys in comparison with girls (2.8±0.6 for boys; 2.6±0.5 for girls; *p* = 0.002). Participants spent 245.7±147.8 min/day in out-of-school SBs with screen time being the most common leisure SB (86.2% of total SB). Children spent 33.9±44.6 min/day in non-screen related sedentary leisure. Children from private schools spent more time in total SB (260.8±151.1 min/day; 231.3±143.2 min/day; *p* = 0.027) and in screen-time SB per day (226.2±142.5 min; 197.9±134.0 min; *p* = 0.039), compared with children from public schools.

**Table 1:** Characteristics of the schools (n = 20).

Variable	n (%)
Type of school	
Public	11 (55%)
Private	9 (45%)
Number of students	
<1000	12 (60%)
≥1000	8 (40%)
Shift school system	
Morning shift only	10 (50%)
Double shift (Morning and afternoon shifts)	6 (30%)
Multiple shifts (Morning, afternoon, and night shifts)	4 (20%)
Monthly school tuition payments (median:29.9)	
$ 0–29.9	11 (55%)
$ 30–139.76	9 (45%)

**Table 2:** Description of participants’ PA, SB, well-being, and nature relatedness by sex and type of school.

Variables	Total	Boys	Girls	t	Public Schools	Privates Schools	t
	n = 1028	n = 495	n = 533		n = 527	n = 501	
	mean (SD)	mean (SD)	mean (SD)		mean (SD)	mean (SD)	
**Age (years)**	10.4 (1.2)	10.4 (1.2)	10.4 (1.2)	0.22	10.3 (1.2)	10.4 (1.2)	-0.95
**Physical Activity (score 1–5)**	2.7 (0.5)	2.8 (0.6)	2.6 (0.5)	-3.58**	2.6 (0.5)	2.7 (0.6)	-1.99
**Total sedentary time (min/day)**	245.7 (147.8)	251.7 (152.1)	240.1 (143.6)	-1.08	231.3 (143.2)	260.8 (151.1)	-2.40*
**Screentime (min/day)**	211.7 (138.9)	218.4 (142.6)	205.5 (135.1)	-1.27	197.9 (134.0)	226.2 (142.5)	-2.21*
**Reading for fun a book or magazine (not for school) (min/day)**	33.9 (44.6)	33.3 (45.3)	34.6 (43.9)	0.48	33.3 (45.5)	34.67 (43.7)	-0.44
**Wellbeing (score 1–5)**	3.7 (0.9)	3.7 (0.9)	3.8 (0.9)	1.08	3.7 (0.9)	3.8 (0.9)	-1.72
**Nature Relatedness (score 1–7)**	4.4 (1.8)	4.3 (1.8)	4.6 (1.8)	2.34*	4.4 (1.9)	4.5 (1.7)	-0.92
**BMI-for-age-z-score (mean score)**	0.5 (1.2)	0.5 (1.2)	0.5 (1.1)	0.02	0.4 (1.2)	0.6 (1.2)	-2.53*

n, Total Number of Individuals; SD, Standard Deviation; RQ, p-value adjusted for clustering.

* p < .05;

** p < .01;

*** p < .0.

Children showed relatively high wellbeing (3.7±0.9; range: 1–5) and nature relatedness (4.4±1.8; range: 1–7) scores. Small differences of nature relatedness mean scores were found between boys and girls, with girls having a higher nature relatedness than boys (4.6±1.8; 4.3±1.8; *p* = 0.03). BMI-for-age-z-score was higher among children enrolled in private schools than those attending public schools (0.6±1.2; 0.4±1.2; *p* = 0.021).

### Associations of nature relatedness with PA and SB

Associations of nature relatedness with PA and SB are shown in Table 3. Nature relatedness was positively associated with PA and non-screen-related sedentary leisure; it was negatively associated with screen time. A higher nature relatedness score was associated with higher PA score (β 0.07; CI 95%: 0.05–0.09; *p* <0.001) and with additional 3.77 minutes spent on non-screen-related sedentary leisure (CI 95%: 0.76–6.68; *p* = 0.016). Likewise, children with a higher nature relatedness score spent 5.59 minutes less on screen time (CI 95%: -10.53–0.65; *p* = 0.029).

**Table 3:** Association of nature relatedness with PA, screen time and non-screen-related sedentary leisure in 9–12 years old school children of Cuenca.

Variables	Physical Activity (score 1–5)			Total sedentary time (min/day)			Screen time SB (min/day)			Non-screen-related sedentary leisure (min/day)		
	β	95% CI	*p*	β	95% CI	*p*	β	95% CI	*p*	β	95% CI	*p*
**Sex (female/male)**	0.17	0.09–0.25	**<0.001**	9.54	-11.03–30.10	0.344	11.93	-7.67–31.55	0.218	-5.19–2.39	-7.43–2.63	0.331
**Age (years)**	- 0.19	-0.04–0.00	0.066	20.15	10.74–29.55	**<0.001**	22.25	13.73–30.77	**<0.001**	-2.10	-4.51–0.31	0.084
**Type of school (public/private)**	0.06	-0.02–0.15	0.140	26.48	4.01–48.95	**0.023**	26.31	3.75–48.87	**0.025**	0.17	-5.90–6.23	0.955
**BMI-for-age-z-score**	0.01	-0.01–0.04	0.273	9.44	2.67–16.19	**0.009**	8.09	2.53–13.66	**0.007**	1.34	-1.64–4.32	0.359
**Well-being**	0.06	0.02–0.10	**0.003**	-5.62	-17.72–6.48	0.343	-5.66	-16.52–5.22	0.290	0.03	-2.87–2.94	0.981
**Nature Relatedness**	0.07	0.05–0.09	**<0.001**	-2.91	-8.52–2.69	0.291	-5.59	-10.53–0.65	**0.029**	2.68	0.55–4.81	**0.016**

Linear regression model was used to examine the independent relationship of nature relatedness with PA, total SB, and screen time SB. Tobit regression model was used to estimate association between nature relatedness and non-screen-related sedentary leisure. All models are adjusted for clustered design of the study, sociodemographic variables, BMI-for-age-z-score, and well-being. Additionally, the models were adjusted for PA when association for SB was examined, and vice versa.

## Discussion

To our knowledge, this is the first study that reports the associations of nature relatedness with PA and SB among school children with different socioeconomic backgrounds and in a LMIC setting. Our results show that children with higher nature relatedness scores spend less time on screen devices, have higher PA levels, and are more often engaged in non-screen-related sedentary leisure, even after adjusting for important covariates.

In the last decade, a growing body of literature has focused on the demographic correlates of PA behavior [38]. Our findings suggest that nature relatedness might be an influential factor for PA among children. Similar associations have been reported in a cross-sectional study in adolescents from Northern Finland [39]. The mechanisms for these findings are complex and need to be explored; the available evidence suggests that positive feelings about nature might motivate green space visits and outdoor recreational activities, which might increase nature connectedness, turning into a positive active lifestyle cycle [14]. In natural environments such as woodlands, open countryside, country parks and urban green spaces, people are more active and more likely to engage in PA and comply with PA guidelines [12]. Objective PA measurements among children showed that PA tends to be more intense in green areas compared to outdoor non-green space [40]. Future studies are needed to identify natural environments that can strengthen children’s nature relatedness in order to improve their PA.

According to our results, children who are less connected to nature spend more time on screen-based activities. This association could be explained by the *displacement hypothesis*, which proposes that the time young people devote to electronic devices displaces time spent on outdoor recreational activities [41] and reduces the opportunities to experience direct contact with nature [42]. Moreover, concerns about the safety of outdoor activities have led caregivers to be more protective, increasing children’s time indoors, reducing children’s PA levels and use of parks [43].

Although screen time has been related with unhealthy outcomes, different types of SB could exert differential effects on health [44]. Non-screen-related sedentary leisure, such as reading for fun, is associated with cognitive and intellectual stimulation and improved academic performance [45]. Moreover, reading for fun in childhood is associated with healthy behaviors in adolescence [45]. In our study, children more connected to nature spent more time on non-screen-related sedentary leisure. No studies have explored this association; we hypothesize that children more connected with nature might enjoy the natural light of natural environments to carry out reading activities.

Models and theories to understand the determinants of SB have mainly focused on environmental factors [46]. Particularly, sedentary lifestyle reduction in children has been associated with availability and accessibility in proximity to green spaces, parks, and recreational facilities [47]. However, built and natural environment aspects are not able to reflect the complex interplay of individual and environmental factors to promote or prevent SB [48]. In addition, home and institutional settings as well as the social and cultural context have been found to be important in the network of associations around SB [49]. Nevertheless, until now, no study has focused on how children’s nature relatedness might influence the time spent on outdoor non-screen-related sedentary leisure and screen devices. More research is needed to understand the connections between declining nature relatedness and increasing screen time SB.

Fostering nature relatedness in children might be a promising strategy to promote PA in natural environments and to reduce screen time, while promoting healthier forms of sedentary leisure, like reading. It has already been proven that nature relatedness in children can be promoted through nature contact and environmental education [12]. In fact, a four-week follow-up after the implementation of an intervention aiming to promote connectedness with nature, showed that the greatest connection with nature retention was sustained at a young age (9–10) compared to older children (11–13) [50]. Experiences such as enjoyment and free play in natural environments, equally available and accessible locations to all socioeconomic groups [51], may have a positive effect on nature connection in children [52]. Moreover, activities that include the natural world such as narrative writing, art, photography, a day in a zoo, or a walk in a natural setting can create a positive change in nature connection [53].

Time outdoors is not the only type of nature contact. Nature-based radio, television, or application programs have been established as indirect forms of contact with nature [54]. Although the use of electronic screen technology has been associated with lower perceived importance of connections to nature and nature-related apps have been related to damage to ecosystems or loss of biodiversity [42], new smartphone apps have been developed to enhance nature relatedness in children [55]. This strategy focusing on the context of environmental education and education for sustainable development has produced a change in students who reported feeling separated from nature [56]. Also, mobile-based interventions have proven to be effective in promoting PA [57]. More research is needed to enhance the use of mobile devices in the promotion of both nature relatedness and PA. Indeed, location-based smartphone games, such as Geogames, have strengthened environmental pro-behaviors and promote PA in natural environments in children by increasing nature relatedness [55]. Research focusing on which type of interactions, direct or indirect contact with nature, has the greatest potential to strengthen a child’s connection to the natural world is necessary.

Our study has several strengths. The main strength is a large, robust, well-powered probabilistic sample. Furthermore, all measurements were collected with calibrated and validated instruments, and performed by qualified personnel. In addition, this project not only considers TV viewing as a proxy for screen time SB but also includes computer and tablet use. Although our findings support the association between nature relatedness with PA and SB, future research is needed to a) incorporate objective measures (accelerometers), b) account for the influence of parental behavior, social support, or educational institutions encouraging less screen time and more outdoor activity [39], and c) examine the function of nature relatedness as a mediator of nature contact and healthy behaviors for future interventions in children [12]. Against these contributions, some limitations should be noted. First, since our study is cross-sectional, causality cannot be inferred in the observed associations as temporality cannot be established. Second, PA and SB might be overestimated or underestimated due to the use of self-reported instruments.

## Conclusions

Low nature relatedness is associated with lower levels of PA, less time spent reading, and more time spent in front of screen devices. Designing and implementing effective strategies for strengthening children’s environmental attitudes might increase children’s natural contact. Fostering nature relatedness might offer an effective strategy to increase children’s PA, limit their screen time, improve their educational outcomes, and prevent negative health outcomes.

## Supporting information

S1 Dataset (XLS)

S1 Fig Residuals vs fitted values graphs for physical activity. (TIF)

S2 Fig Residuals vs fitted values graphs for total sedentary behavior. (TIF)

S3 Fig Residuals vs fitted values graphs for screen time. (TIF)

S4 Fig Residuals vs fitted values graphs for non-screen-related sedentary leisure. (TIF)
