# Supplementary figures and images for: Nature relatedness as a potential factor to promote physical activity and reduce sedentary behavior in Ecuadorian children
Source: PLoS One. 2021 May 20;16(5):e0251972. doi: 10.1371/journal.pone.0251972 (PMC8136842; doi:10.1371/journal.pone.0251972)

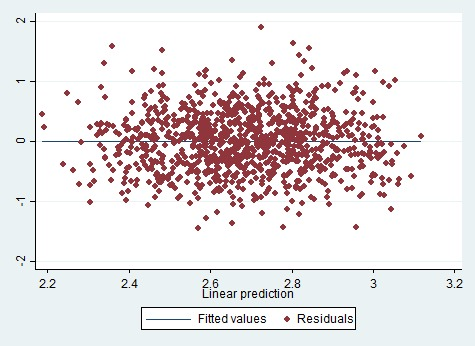

Supplement: S1 Fig — (TIF) [file pone.0251972.s002.tif]

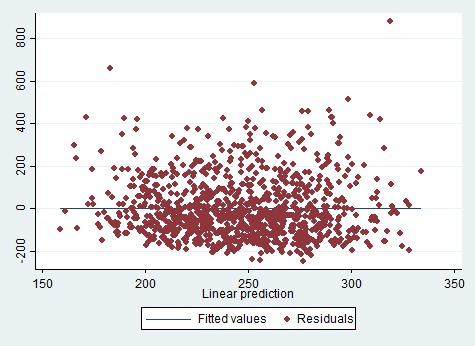

Supplement: S2 Fig — (TIF) [file pone.0251972.s003.tif]

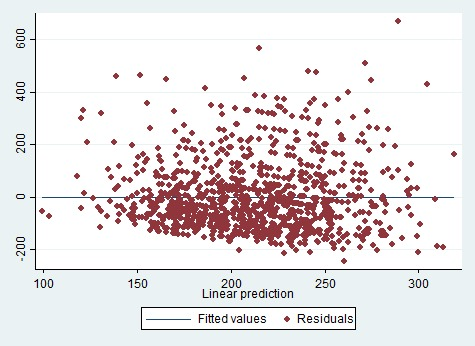

Supplement: S3 Fig — (TIF) [file pone.0251972.s004.tif]

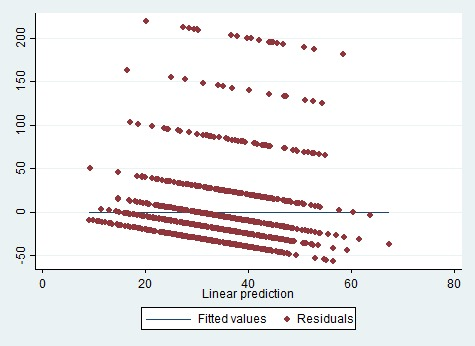

Supplement: S4 Fig — (TIF) [file pone.0251972.s005.tif]
